# Effects of Smart City Construction on the Quality of Public Occupational Health: Empirical Evidence From Guangdong Province, China

**DOI:** 10.3389/fpubh.2021.769687

**Published:** 2021-10-21

**Authors:** Hao Cheng, Fan-Fan Wang, Da-Wei Dong, Ji-Chao Liang, Chun-Fen Zhao, Bei Yan

**Affiliations:** ^1^Key Laboratory of Environment Change and Resources Use in Beibu Gulf Ministry of Education, Nanning Normal University, Nanning, China; ^2^College of Economics and Management, Nanning Normal University, Nanning, China; ^3^School of Public Administration, South China University of Technology, Guangzhou, China; ^4^The Academy of Macroeconomic Research of Guangxi Zhuang Autonomous Region, Nanning, China; ^5^The Folk Culture and Art Research Institute of Guangxi Zhuang Autonomous Region, Nanning, China; ^6^Nanning United Innovation Venture Capital Co., Ltd., Nanning, China; ^7^Department of Mathematic and Computer Technology, Guilin Normal College, Guilin, China; ^8^School of Government, Universiti Utara Malaysia, Sintok, Malaysia

**Keywords:** smart city construction, quality of public occupational health, policy effect, difference-in-difference model, Guangdong Province

## Abstract

This article takes the Guangdong Province of China as the research object and uses the difference-in-difference model to evaluate the impact of smart city construction on the quality of public occupational health and intercity differences. The obtained results show that smart city construction significantly improves the quality of public occupational health, and it is still valid after a series of robustness tests. The effect of this policy is stronger in cities that belong to the Pearl River Delta region or sub-provincial level cities. This study indicates that the central government should improve the pilot evaluation system and the performance appraisal mechanism of smart cities from the perspective of top-level design during the process of promoting smart city construction, which aims to correctly guide local governments to promote the construction of smart cities. To achieve the full improvement effect of smart city construction on the quality of public occupational health, local governments should implement smart city strategies in a purposeful and planned way according to the actual situation of the development of the jurisdiction.

## Introduction

This article aims to investigate whether smart city construction (measured by the smart city construction pilot policy since 2012) has an impact on the occupational health quality of the public (measured in reverse by the death rate of 100 million yuan of GDP of occupational safety accidents and death number of occupational safety accidents). The strong aspiration and the demand for a better life of people in all countries, especially in developing countries, have injected endless impetus into urbanisation. Urbanisation has greatly promoted the development of society, economy, and science and technology. However, at the same time, it has brought great challenges to the sustainable development of human beings, and it also brings new challenges to the Earth's capital environment (1). Urban issues (such as traffic, environment, housing, employment, public safety, and health) are prevalent in cities worldwide. These problems have begun to seriously affect the sustainable development of the economy and the quality of life of human beings (2). With the gradual maturity and wide application of internet technology, human beings have stepped into the era of knowledge networks. Knowledge innovation and application have become the key factors leading to economic and social development and progress (3). Especially since the beginning of the 21st century, as the global urban population continues to increase, as a solution to the economic and social problems brought by unprecedented rapid urbanisation, smart city construction has become a trend of the times and plays an important role in promoting and leading the healthy development of cities.

In 2009, IBM proposed the concept of “smart cities” for the first time, that is, making full use of information and communication technology to intelligently perceive, integrate, analyse, and respond to the needs and related activities of cities in the exercise of market regulation, economic regulation, social management, and public service functions (4). Once the concept of a “smart city” was put forward, foreign countries, especially developed countries in Europe and the United States, actively put it into practise, and the construction system of smart cities has become increasingly perfect. In the United States, Europe and developed Asian countries, including (Japan, South Korea, and Singapore), smart city construction projects are in full swing and have achieved remarkable results (5, 6). After more than 40 years of rapid development through reform and opening up in China, urban issues (such as serious environmental pollution, frequent safety accidents, traffic congestion, and housing shortage) have become increasingly prominent. In particular, the quality of public occupational health is suffering from a huge impact due to problems such as imperfect regulations and standards, lax safety supervision and law enforcement, imperfect supervision systems and mechanisms, weak safety foundation, and weak emergency rescue capacity. Therefore, driven by the Internet of Things, Internet and cloud computing-based information technology, smart city pilot policy of China has begun to develop.

However, in the context of China, smart city construction needs to face special political, economic, and social environments. Can the pilot policies of smart city construction improve the quality of public occupational health? Based on the balanced panel data of 20 cities in Guangdong Province, China, this article empirically tests the impact of smart city construction on the quality of public occupational health. The main marginal contribution is reflected in the following three aspects. First, this article uses the data from 20 cities in the Guangdong Province from 2001 to 2019 for the first time and discusses the influencing factors of public occupational health quality from the perspective of smart city construction, which has important practical significance for how to effectively improve the public occupational health at present. Second, this article uses the smart city pilot as a “quasi-natural” experiment, effectively overcomes the estimation errors of traditional research methods, and conducts a multidimensional robustness test, which aims to be more accurately analysing the impact of smart city construction on the quality of public occupational health. Third, from the perspective of administrative levels and regional differences, this paper further analyses the factors that may affect the policy effect of smart city construction and provides some enlightenment for the reasonable layout and continuous improvement of smart city construction policies.

The structure of this article is as follows: section Literature Reviews the existing literature and section Smart City Construction and the Quality of Public Occupational Health carries on the theoretical analysis and proposes a hypothesis. Section Methodology introduces the empirical method applied. Section Data shows empirical data and variables. Section Empirical Results analyses our empirical results. Section Conclusions presents the concluding remarks.

## Literature Review

Research on “smart cities” has its origins in improving the quality of life of residents. This involves developing new ways and measures to improve urban public management and provide public services (7). There are many similarities between the concepts of smart cities and digital cities (8, 9), but smart cities are considered to be a broader concept that aims to unite, facilitate, and encourage the dissemination of information to improve the quality of public life. All of these are different due to the different cooperative relationships among urban stakeholders (10). Different governance methods of smart cities lead to vague and inconsistent views on the concept of smart cities (11). Nevertheless, most scholars agree that smart cities are a broad concept. By mobilising various forces and applying interdisciplinary knowledge, smart city subjects seek innovative and sustainable governance solutions to solve many problems of urbanisation. In other words, smart cities can be described as regions presented in a forward-looking way.

In 2014, the Development and Reform Commission of China, together with seven ministries and commissions, issued the “Guiding Opinions on Promoting the Healthy Development of Smart Cities,” which put forward six requirements for the construction of smart cities, including convenient public services, fine urban management, livable living environment, intelligent infrastructure, and long-term network security. As one of the goals of smart city construction and high-quality economic development, the influence of these factors on the quality of public occupational health has undoubtedly become one of the key areas for scholars to study (12). From the perspective of enterprise management, Chen et al. (13) performed a statistical analysis of 410 major gas explosion accidents from 1980 to 2000 and determined that the proportion of accidents caused by human factors exceeded 96.59%, indicating that coal mine accident is a management problem rather than an unsolvable technical problem. The “Swiss Cheese Model” proposed by Reason (14) established the link between unsafe behaviours, unsafe conditions, and organisational management behaviours, and attributed the cause of accidents to the superposition of individual mistakes and organisational management mistakes.

The economic policy also plays an important role in the quality of public occupational health. Inadequate protection of property rights is one of the key factors. Mine accidents in China mainly occur in small and medium-sized coal mines dominated by township coal mines because they lack clear property rights, stable expectations, adequate safety input, and perfect occupational health conditions (15). Cohn and Wardlaw (16) used corporate data in the United States and determined that an excessive corporate debt ratio would lead to insufficient investment and more work-related accidents. Nie and Zhao (17) used the panel data of coal enterprises of China from 2001 to 2006 and determined that the corporate debt ratio significantly increased the number of deaths in mining accidents and greatly affected the quality of the occupational health of workers. In addition, the low level of production technology, lack of safety measures, backward health concepts, and lack of property rights protection systems are also the key factors that threaten the quality of occupational health (18).

The last aspect concerns the impact of political factors on the quality of public occupational health. The role of government regulation is first emphasised. Lewis and Alford (19) believed that the occupational health of workers was determined by the enforcement of government regulatory policies. Using the legislation of the United States in 1941 and 1969, they tested the relationship between enforcement and coal mine mortality and determined that the strengthening of government regulation could help reduce the number of mining deaths, thus, improving the quality of occupational health of workers. However, Grey and Scholz (20) determined that the safety regulatory agencies in the United States were ineffective when using the data, thus, proposing a theory to refute regulation. Appropriate hard regulation can, indeed, improve the quality of occupational health (21), but overly strict regulatory policies cannot achieve the purpose of protecting workers and improving safety (22). Despite the lack of binding legal force, soft regulation is considered an important governance tool to improve the quality of occupational health, especially when combined with hard regulation (23, 24). Some scholars also emphasise the influence of political connexions. Fisman and Wang (25) used data on Chinese-listed companies from 2008 to 2013 to analyse the relationship between political affiliations and corporate accidents and determined that the death rate of politically affiliated companies was two to three times higher than that of companies without political affiliations. Jia and Nie (26) regard the “centralisation—decentralisation—centralisation” of key state-owned coal mine regulators in China from 1995 to 2005 as a “quasi-natural” experiment and find that decentralisation of coal mine supervision leads to more collusion between local governments and coal mining enterprises, resulting in higher mortality and lower occupational health quality.

From the development experience of various countries, the technical support provided by smart cities can more effectively optimise the layout of urban development. In particular, combined with the specific application of urban planning and construction, occupational health concepts, work safety technology, and platforms should be effectively combined to compensate for the safety accidents caused by technology, management, and personnel so as to effectively improve the quality of public occupational health. Therefore, it is particularly important to accurately evaluate the impact of smart city construction on the quality of public occupational health from the perspective of empirical testing.

## Smart City Construction and the Quality of Public Occupational Health

From the perspective of theoretical research, accident cause theory has changed from the early single factor theory to multiple-cause theory, in which the interaction between man, machine, and environment is the cause of the accident. The health factors in the workplace of China include management, human, material, and environment (27). The fundamental reason for the relatively low occupational health quality lies in the management errors in the process of internal and external management of enterprises. Among them, the vulnerability of engineering technology, management systems, and personnel quality are the main reasons for the low health quality in the workplace. The smart city construction can compensate for the abovementioned defects in the following four ways to improve the quality of public occupational health.

Smart city construction reduces the transaction cost of enterprises and improves the health management ability of employees. According to the transaction cost theory proposed by Coase, transaction cost refers to the cost of obtaining accurate market information, negotiation, and contract costs (28). Smart can expand limited rational cost, inhibit opportunism behaviours, reduce the uncertainty, and enhance the role of trading frequency to drive the enterprise transaction cost reduction (29), to change the enterprise organisation form, to influence enterprise production, management, marketing, and the purchasing mode, to significantly strengthen the internal management, and to improve production efficiency (30), which helps improve the occupational health of enterprises. Smart city construction optimises the government service function and improves the efficiency of the occupational health quality management of the government, which realises the wisdom of economy, transportation, environment, life, government service, and other fields through the accumulation of big data and the development of emerging technologies (31). Especially in the field of government services, smart city construction realises the reengineering of government processes and the transformation of government functions, which help to improve the governance capacity and the management level of the government and provide capacity guarantees for the quality of public occupational health. Smart city construction can effectively alleviate the disadvantages of collusion between the government and enterprises, which improves the ability of the government to supervise public occupational health. The literature on government-enterprise collusion points out that the existence of information asymmetry between the central government and local governments and the economic performance appraisal system under political centralisation leads to the formation of collusion between local governments and enterprises (26). Smart city construction can contain many people in the government and enterprises and embed input control, processing control, and output control into the system to effectively reduce the opportunistic behaviours, such as mutual use, collusion, and collusion between local governments and enterprises caused by the information asymmetry between the central government and local governments. Through the construction of smart cities, the government can effectively improve its decision-making and execution; enterprises can effectively improve operational efficiency and reduce transaction costs, and the public can enjoy a more convenient, comfortable, and healthy life. Based on the abovementioned analysis, this article proposes the hypothesis of the first level:

- Hypothesis 1: Smart city construction can improve the quality of public occupational health.

Urban heterogeneity and diversity are important sources to promote economic growth, which are reflected in the integration force of urban resource absorption, production and consumption capacity, and transaction costs (32). For industries that rely on technology research and new technology, smart city construction policies may differ greatly due to differences in factor endowments in different regions. Because the institutional framework of regional development is the key factor that restrains the actual effect of policy, soft constraints (such as economic structure, education system, and policy reform) will restrict the effect of capital and other hard inputs. The actual development of smart cities in China is influenced by politics, economy and geographical location. Different regions have great differences in resources, technologies, and public occupational health management policies, which may affect the effect of smart city construction on improving public occupational health. Among them, differences in administrative levels and regions are the fundamental causes of different institutional frameworks.

By studying the relationship between government and economic development in the Yangtze River Delta region of China, Shuih (33) believes that the rapid economic development of China is due to the resource advantages brought by the change and adjustment of the administrative level of the government and the promoting role of the political system in opening and innovation. Cities with higher administrative levels are distributed in areas with convenient transportation, complete infrastructure, and a high level of openness. Their development advantages promote the accumulation of factor stocks, which aims to mobilise resources for urban innovation construction and promote and improve the construction system of smart cities. At the same time, cities with higher administrative levels have more power of administrative examination and approval, a higher financial scale, and more central strategic policy status. The abovementioned characteristics of cities at high administrative levels are likely to affect the policy effect of smart city construction.

According to the actual development of Guangdong Province, there are significant differences in economic, political, and social development between the Pearl River Delta region (PRD) and the non-Pearl River Delta region (non-PRD) due to the influence of policy support, resource endowment, geographical location, and natural conditions. Statistics released by the “Guangdong Statistical Yearbook” in 2019 show that PRD, which accounts for 20% of the administrative area of Guangdong Province, accounts for more than 80% of the GDP of the province, while non-PRD, which accounts for 80% of the administrative area, accounts for less than 20% of the GDP of the province, indicating a significant imbalance in economic development, which will certainly have an impact on the policy effect of smart city construction. PRD itself, in terms of resource acquisition, technical support, and preferential policy, has many advantages over non-PRD of weak infrastructure, coupled with a single industrial structure, low marketisation degree, and soft environment, such as an education concept-relative lag. It is easy to restrict the policy effect of smart city construction. Based on the abovementioned analysis, this paper proposes a second hypothesis to be tested.

Hypothesis 2: Compared with general prefecture-level cities, the policy effect of smart city construction on the quality of improving public occupational health is more obvious in sub-provincial cities.
Hypothesis 3: Compared with non-PRD, the policy effect of smart city construction on improving the quality of public occupational health is more obvious in PRD.

## Methodology

China put forward the concept of smart city construction in 2009 and started the first batch of pilot work on smart cities in 2012. The second and third batches of pilot work were performed in 2013 and 2015, which provided an opportunity for a quasi-natural experiment. In this article, cities with smart city pilot units are treated as the treatment group; cities without smart city pilot units are treated as the control group, and the difference of differences model is realised through a two-way fixed effect to test the impact of smart city construction on the quality of public occupational health. Because the pilot work of smart city construction does not take place in the same year, a more extensive DID model is adopted, and the time when the pilot is set up in the corresponding year is used as the time point when the policy takes place.

Considering that, among the 21 prefecture-level and abovementioned cities in the Guangdong Province, Guangzhou, Shenzhen, Foshan, Zhaoqing, Dongguan, Zhongshan, and Heyuan, all take towns or counties as the pilot units, while Zhuhai takes cities as the pilot units; to avoid possible deviations, the sample of Zhuhai is deleted. Therefore, the treatment group of this paper includes seven samples of cities, and the control group includes 13 samples of cities (see Table 1).

**Table 1:** Details of the cities of treatment and control group.

**Time**	**Treatment group**	**Control group**
2012	Guangzhou, Shenzhen, Foshan,	Jiangmen, Zhaoqing, Huizhou, Shantou, Chaozhou, Jieyang, Shanwei, Maoming, Yangjiang, Yunfu, Shaoguan, Qingyuan, Meizhou
2013	Zhaoqing, Dongguan, Zhongshan	
2015	Heyuan	

**Figure 1:**
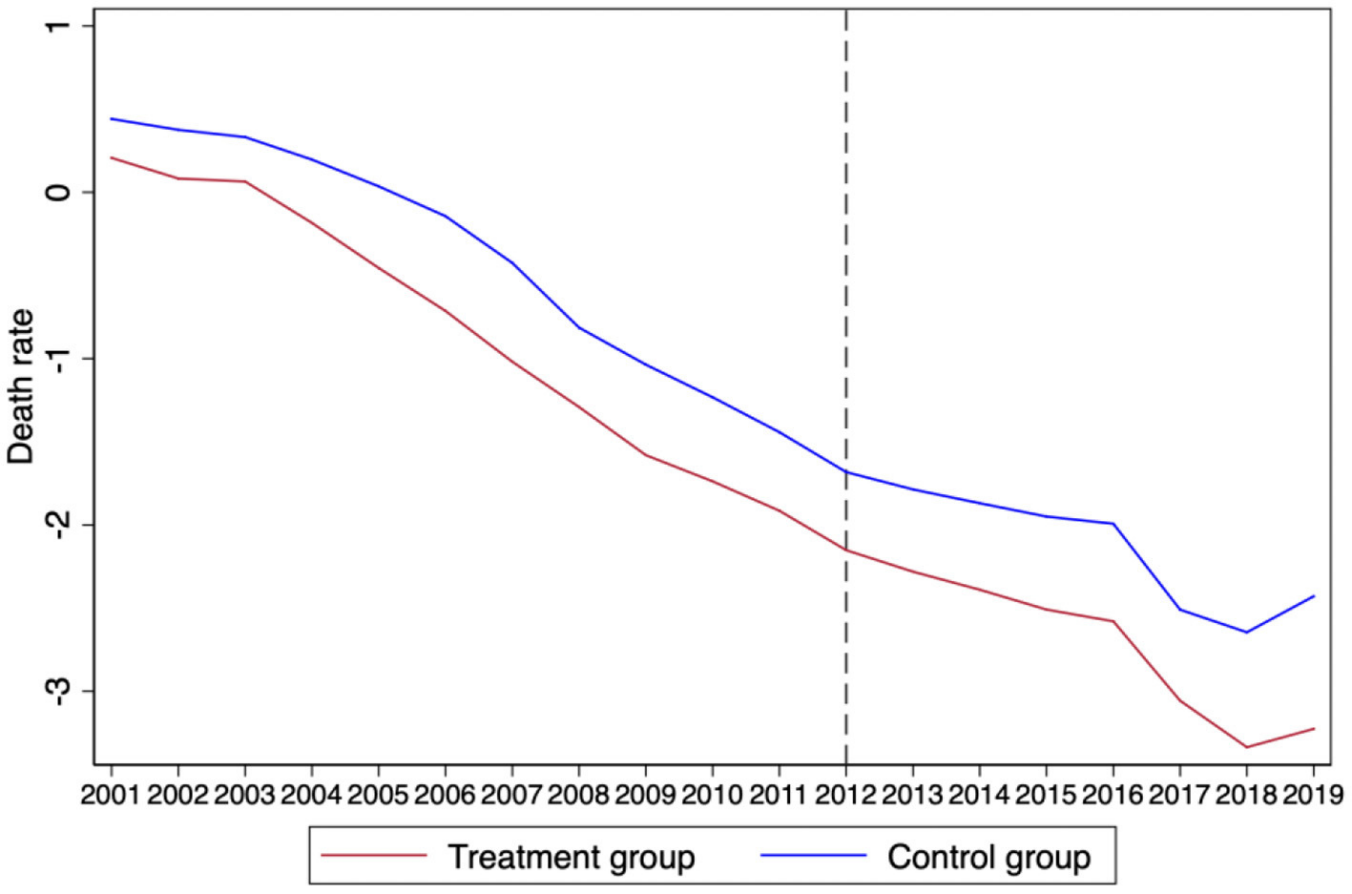
The result of parallel trend (death rate).

**Figure 2:**
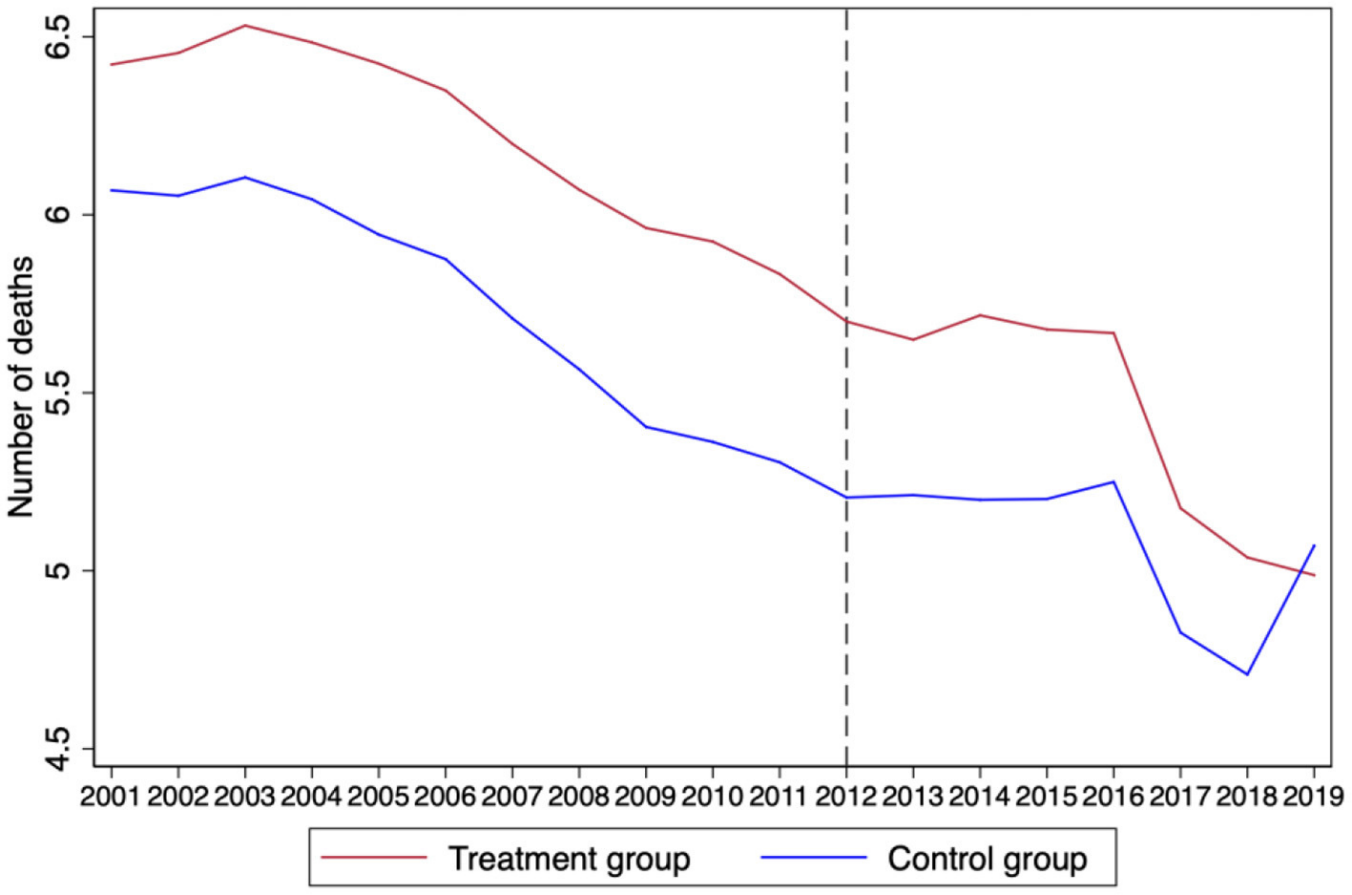
The result of parallel trend (number of death).

The most important prerequisite for using the DID model is the assumption that the processing group and the control group satisfy parallel trends before the policy impact (34). The mean value of the treatment group and the control group over the years was used to obtain the time trend diagram as shown in Figures 1, 2, which shows that the changing trend of the death rate and the number of deaths before 2012 were the same, and, after 2012, the changing trend of the treatment group significantly decreased compared with the control group. Therefore, we preliminarily judged that the treatment group and the control group met the conclusion of trend consistency.

To test the impact of smart city construction on the quality of public occupational health and test Hypothesis 1, we achieve the purpose of the difference of differences model by controlling two-way fixed effects, and construct the following econometric model (35):


(1) $$\begin{array}{r} Y_{it}=\beta_{0}+\beta_{1}Wise_{it}+\sum_{it}Control_{it}+\mu_{i}+\delta_{i}+\varepsilon_{it} \end{array}$$

where *i* represents the city and *t* represents the year; μ_*i*_ indicates city-fixed effects, δ_*i*_ indicates year-fixed effects, and the dependent variable *Y*_*it*_ represents the quality of public occupational health. *Wise*_*it*_ represents whether smart city construction is performed in city *i* in year *t*, and it takes one if city *i* has smart city pilot units in year *t* and the years after and zero otherwise. *Control*_*it*_ represents a series of control variables, which includes the economic development level (*ln*_*gdp*), industrial structure (*ln*_*ind*), fixed asset investment level (*ln*_*inv*), population size (*ln*_*pop*), urbanisation level (*In*_*urb*), and research and development level (*ln*_*rad*). ε_*it*_ is the random perturbation term. For the abovementioned model, this article pays more attention to the estimate of the coefficient β_1_, which measures the effect of smart city construction on the quality of public occupational health. If the establishment of smart city construction does improve the quality of public occupational health, then the coefficient of β_1_ should be significantly negative.

Since the smart city pilot policy has been implemented in batches since 2012, rather than all at once, the status of a city in the treatment group or the control group may change. For example, if a city was a non-pilot city in 2012 (control group), it may become a pilot city in 2013 (treatment group). In this case, it is more reasonable and accurate to use the event analysis method to detect the parallel trend, which is also widely recognised and used in the literature related to the DID model, compared with the method of drawing the trend chart using the average value of the treatment group and the control group, respectively. Drawing on the practise of Beck et al. (36), this paper sets the following econometric model:


(2) $$\begin{array}{r} Y_{it}=\beta_{0}+\sum_{m=k,m\neq -1}^{M}Wise_{it,k}\;*\;\beta^{k}+\sum_{it}Control_{it}\\ \;+\mu_{i}+\delta_{i}+\varepsilon_{it} \end{array}$$

where *Wise*_*it, k*_ is a series of dummy variables representing the treatment status at different periods. The dummy for *m*= −1 is omitted in Equation (2) so that the post smart city construction effects are relative to the period immediately before the launch of the policy. The parameter of interest β^*k*^ estimates the effect of smart city *m* year after the construction. Because the pilot policy for smart city construction is not performed in all cities at the same time, *k* represent different years for different cities. Intuitively, the coefficient β^*k*^ measures the difference in the quality of public occupational health between cities under the pilot policy performed for smart city construction and, otherwise, in period *k* relative to the difference 1 year before the pilot policy was performed for smart cities. We expect smart city construction to improve the quality of public occupational health, with β^*k*^ being positive when *k* ≥ 0. If the parallel trend assumption holds, β^*k*^ would be close to zero when *k* ≤ −2.

We have pointed out that the effect of smart city construction on the quality of public occupational health may be affected by administrative level differences and regional differences. We construct the following model to test Hypothesis 2:


(3) $$\begin{array}{r} Y_{it}=\beta_{0}+\beta_{1}Wise_{it}+\beta_{2}Wise_{it}\;*\;City_{it}+\beta_{3}City_{it}\\ \;+\sum_{it}Control_{it}+\mu_{i}+\delta_{i}+\;\varepsilon_{it} \end{array}$$

In Formula 3, *City*_*it*_ represents the city characteristic variables, including the following two aspects. The first one is the administrative level difference (*SUB*_*it*_), which denotes whether city *i* is a sub-provincial city, and it takes one if city *i* is a sub-provincial city and zero otherwise. The second is the regional difference (*RGO*_*it*_), which denotes whether city *i* belongs to PRD, and it takes one if city *i* belongs to PRD. Otherwise, it takes zero. What we care about is whether the coefficient of β_2_ is significant.

## Data

Located in the south of China, Guangdong Province is a very important economic province in China and a pioneer of reform and opening up. Since 1989, the GDP of Guangdong has been the first in China, and it has become the largest economic province in China. Its economic aggregate accounts for 1/8 of the national total, which has reached the level of upper-middle-income countries. This paper uses the sampling data of Guangdong Province, which mainly considers two aspects. First, the data availability, because most cities did not release occupational health management-related indicators, but the “Guangdong Statistical Yearbook” and “Statistical Bulletin on National Economic and Social Development” of 20 cities released the related indicators. The second, the representativeness of the sample. Since the new century, the production safety accident types involved in transportation and warehousing, trade and manufacturing, construction and other fields and the economic and social development level of PRD and the eastern, western, and northern of Guangdong Province have obvious imbalances, which are similar to those of the whole country of China. We collect balanced panel data from 2001 to 2019 for 20 cities at the prefecture level and above in the Guangdong Province of China. All data are obtained from the “Guangdong Statistical Yearbook” and the “Statistical Bulletin of National Economic and Social Development” of each city.

### Dependent Variables

From the perspective of absolute and relative indicators, this paper uses a death rate of 100 million yuan of GDP for occupational safety accidents (death rate, *Ln*_*rate*) and the death toll of occupational safety accidents (number of deaths, *Ln*_*death*) to reverse the estimate of the quality of public occupational health from the absolute and relative numbers.

### Independent Variables

The core independent variable of this paper is smart city construction. China set up the first batch of smart city pilot units in 2012, followed by the second and third batches in 2013 and 2015, respectively. In this article, the core independent variable denotes whether smart city construction is performed in city *i* in year *t*, and it takes one if city *i* has smart city pilot units in year *t* and the years after and zero otherwise.

### Regional Characteristic Control Variables

To more accurately assess the impact of smart city construction on the quality of public occupational health, we incorporated six variables (including the economic development level, industrial structure, fixed asset investment level, population size, urbanisation level, research and development level, and government transition effect) into the control variables. The first variable considered is the level of economic development. Safety management investment is insufficient and is the important factor that brings about safety accidents to take place frequently. The economic development level is the higher area, and the safety management investment and the technical level are higher (37). This article controls the impact of the economic development level on the quality of public occupational health by adding *per capita* GDP. The second variable considered is industrial structure. Safety accidents have obvious industrial characteristics. Both the mining industry and the construction industry belong to industries with poor safety status, and the fatality rate of the mining industry is much higher than the social average. In this article, the ratio of the total output value of the tertiary industry to the total output value of the secondary industry is used to measure the industrial structure. The third variable considered is an investment in fixed assets. The improvement of investment in fixed assets can help enterprises upgrade equipment and technical transformation, thus improving the safety management level of enterprises and reducing the death level of safety accidents (38). This article uses fixed assets investment to measure. The fourth variable considered is population size. We controlled for the effect of population on the quality of public occupational health by adding the variable of population size. The fifth variable considered is the level of urbanisation. The higher the level of urbanisation, the higher is the public demand for health, which is conducive to promoting the improvement of the health level. Therefore, the improvement of the urbanisation level may lead to the improvement in the quality of public occupational health. This article uses the proportion of the urban population in the total population of each city for the measurement. The sixth variable considered is the level of research and development. A low level of production technology is an important factor leading to the low quality of occupational health (39), and the improvement of the research and development level is conducive to reducing the occurrence of occupational safety accidents and improving occupational health quality. In this article, the proportion of research and development personnel in the total population was measured (40, 41). To ensure the accuracy of the measurement results, this paper performed logarithmic processing of the indicators. Descriptive statistics of related variables are shown in Table 2. The number of observations in the treatment group is 43, accounting for approximately 11.32% of the total number of samples.

**Table 2:** Descriptive statistics of key variables.

	**Total**			**Treatment group**			**Control group**		
	**Obs**.	**Mean**	**Sd**.	**Obs**.	**Mean**	**Sd**.	**Obs**.	**Mean**	**Std**.
ln_rate	380	−1.258	1.194	43	−2.892	0.671	337	−1.049	1.080
ln_death	380	5.608	0.753	43	5.429	0.689	337	5.656	0.762
ln_pgdp	380	10.15	0.867	43	11.404	0.529	337	9.990	0.765
ln_ind	380	4.474	0.339	43	4.658	0.441	337	4.451	0.317
ln_inv	380	5.986	1.226	43	7.642	0.686	337	5.774	1.114
ln_pop	380	5.887	0.530	43	6.049	0.461	337	5.867	0.535
ln_urb	380	3.911	0.411	43	4.350	0.312	337	3.854	0.388
ln_rad	380	8.501	1.716	43	10.694	1.355	337	8.221	1.549

## Empirical Results

The main empirical results of smart city construction policy evaluation are divided into three parts. The DID model is first used to estimate the impact of smart city construction on the quality of public occupational health. Then, the heterogeneity of policy effects is discussed. Finally, the identification hypothesis test and robustness test are performed, respectively, to eliminate the estimation bias caused by the omission of variables.

### Baseline Regression Result

Hypothesis 1 of this article is that the construction of smart cities is conducive to promoting the quality of public occupational health, and the death rate and the number of deaths are used as reverse indicators to measure the quality of public occupational health. Table 3 reports the baseline regression results based on Formula (1). Without controlling other factors, columns (1) and (2) show that the coefficient of smart city construction is negative, and the significance levels are 1% and 5%, respectively, indicating that smart city construction is powerful in promoting the quality of public occupational health. This article continues to introduce six control variables to obtain columns (3) and (4). The obtained results show that the coefficients of smart city construction are −0.276 and −0.179, respectively, and both pass the significance level of 1%, indicating that smart city construction reduces the death rate and the number of deaths by 0.276 and 0.179 units, respectively. Based on the results, it can be found that smart city construction has a significant positive effect on the quality of public occupational health. Hypothesis 1 is confirmed.

**Table 3:** Baseline regression results.

	**ln_rate**	**ln_death**	**ln_rate**	**ln_death**
	**(1)**	**(2)**	**(3)**	**(4)**
Wise	−0.150***	−0.114**	−0.276***	−0.179***
	(0.056)	(0.050)	(0.057)	(0.053)
ln_pgdp			−0.422***	−0.262***
			(0.096)	(0.090)
ln_ind			−0.018	−0.124**
			(0.065)	(0.061)
ln_inv			−0.221***	0.009
			(0.056)	(0.052)
ln_pop			−0.147*	−0.270***
			(0.075)	(0.070)
ln_urb			−0.182**	−0.017
			(0.092)	(0.086)
ln_rad			−0.047**	0.015
			(0.020)	(0.019)
Region FE	Yes	Yes	Yes	Yes
Year FE	Yes	Yes	Yes	Yes
Constant	0.359***	6.192***	7.252***	10.583***
	(0.056)	(0.050)	(1.209)	(1.129)
*R* ^2^	0.954	0.811	0.960	0.825
*N*	380	380	380	380

***, **, and *, respectively, indicate significance at the 1, 5, and 10% level. Standard error in parentheses.

From the obtained results of the control variable, the economic development level, the level of investment in fixed assets, the population size, the urbanisation level, the research and development level effect for the accident mortality have a significant negative effect except for industrial structure, but, in terms of deaths, only the economic development level, industrial structure, and population scale have a significant effect.

### Parallel Trend Test

In view of the DID model used above to estimate the impact of smart city construction on the quality of public occupational health, a key premise for obtaining a nonbiased regression result is the assumption that parallel trends need to be satisfied between the treatment group and the control group. If the treatment and the control groups had different time trends before being affected by the smart city pilot policy, the changes in the quality of public occupational health may not be caused by the smart city pilot but by the time trend before the policy pilot. With the help of the framework of event analysis, this paper examines again whether there is a parallel trend in the changes of the quality of public occupational health quality in the treatment and control groups before the smart city construction policy.

**Figure 3:**
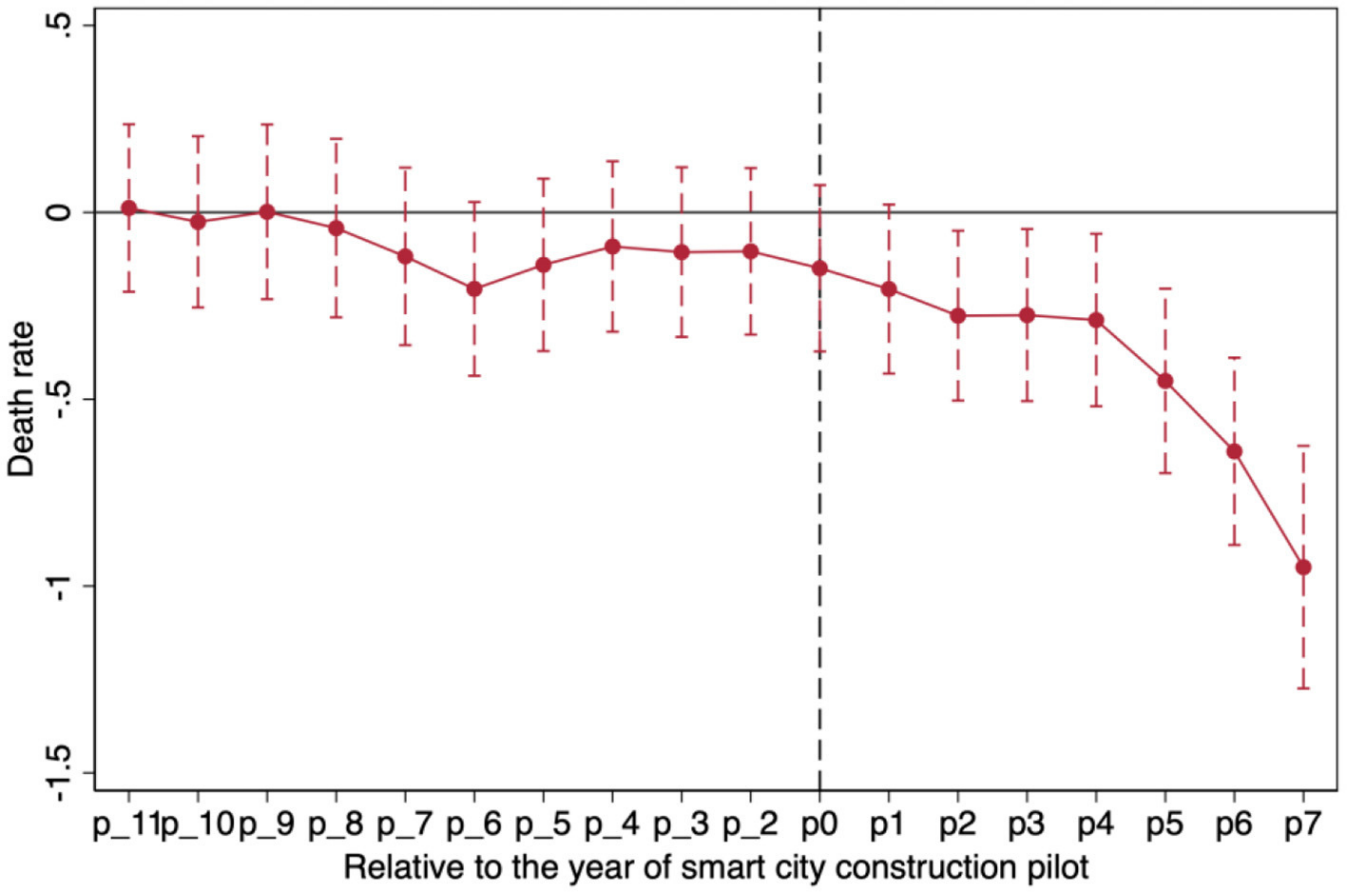
The result of dynamic effects (death rate).

**Figure 4:**
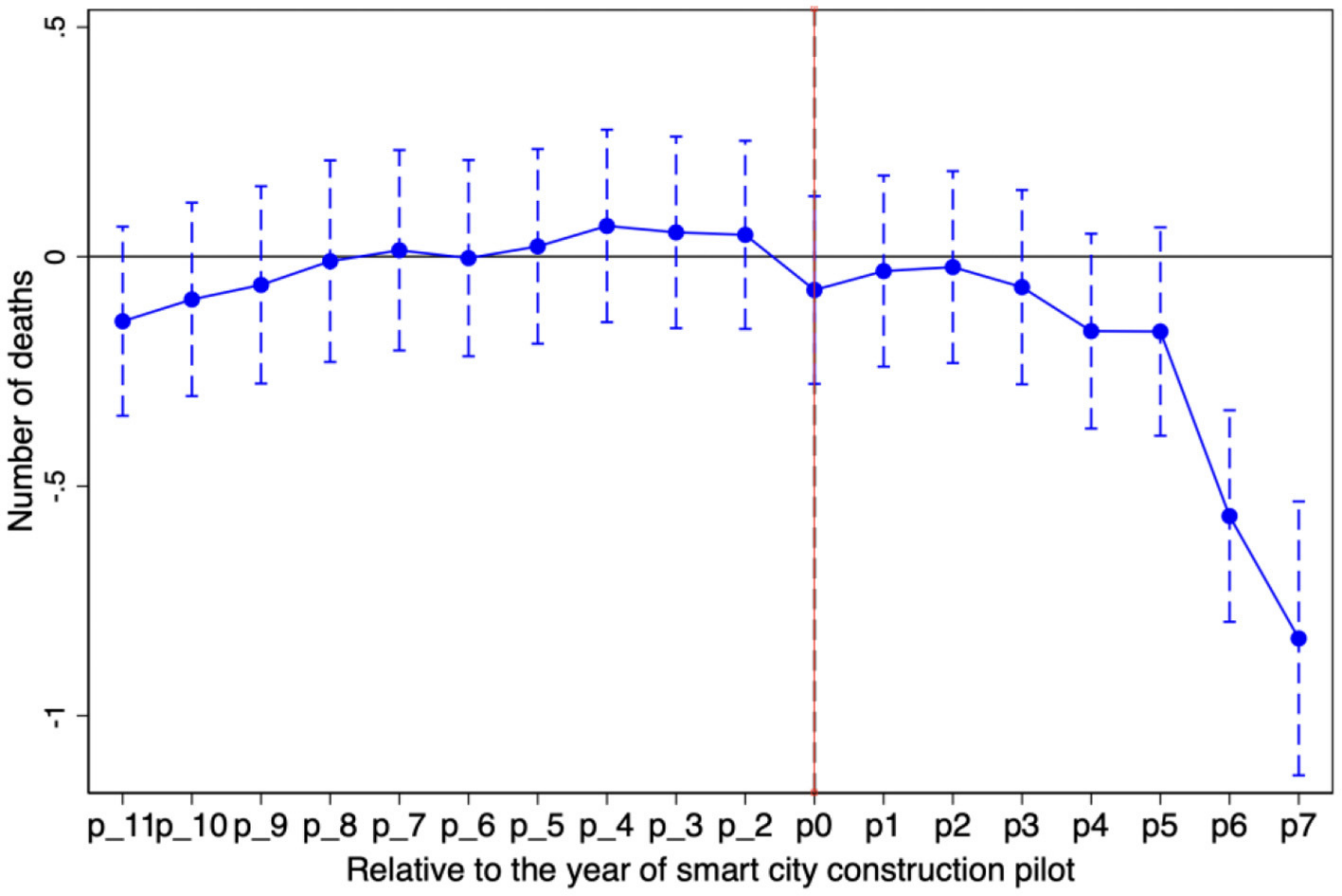
The result of dynamic effects (number of deaths).

Figures 3, 4 report the estimated dynamic effects of coefficients with dependent variables of the death rate and the death number, respectively. Figures 3, 4 show that the coefficient of smart city construction has not been estimated at the 5% significance level before the pilot year virtual variable, which indicates that the treatment and control groups were verified to meet parallel trend assumption. Namely, the smart city construction pilot-relative control group after the treatment group death rate significantly decreased is the result of smart city construction rather than the result of differences in advance. Meanwhile, the impact of smart city construction pilots on the quality of public occupational health has good policy sustainability.

### Robustness Tests

#### PSM+DID

Although the DID model can well identify the net effect of smart city construction policy, it cannot overcome the problem of sample selection bias. Because it is not random whether a city is identified as a smart city construction unit, it may cause regression bias if we directly estimate nonrandom samples. In addition, part of the difference in the quality of public occupational health between the treatment group and the control group may be due to other unobservable factors and factors that do not change over time, and our direct estimation of nonrandom samples may lead to the problem of bias.

**Figure 5:**
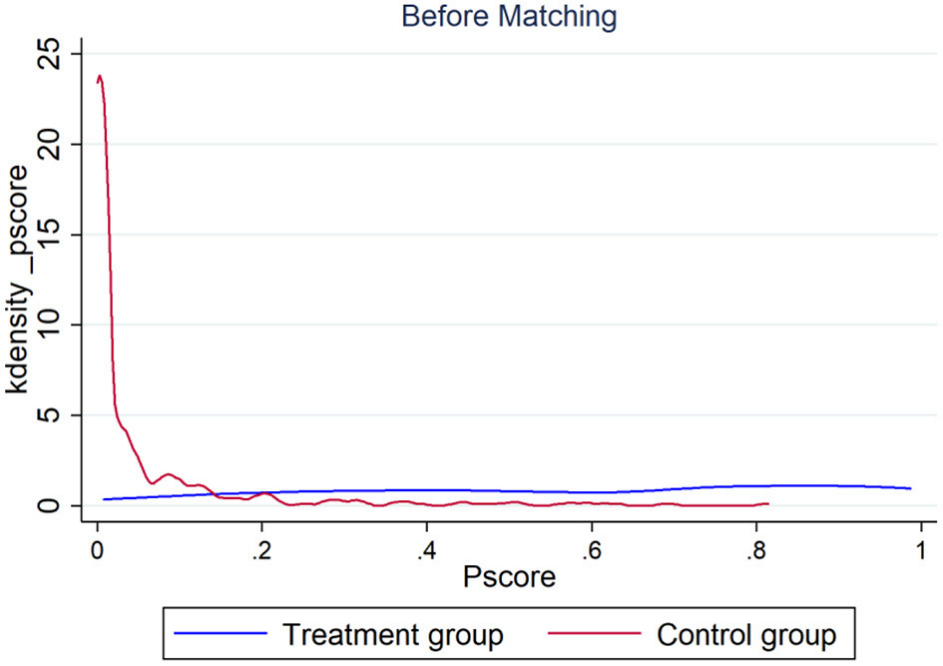
Kernel density distribution of the propensity score before matching.

**Figure 6:**
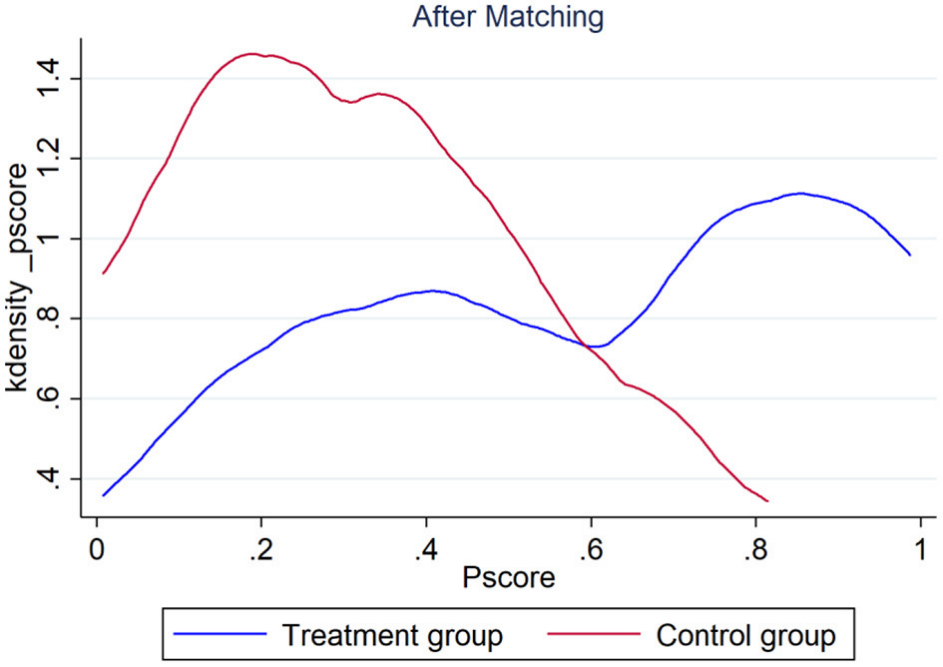
Kernel density distribution of the propensity score after matching.

To test the robustness of the benchmark regression results and ensure the reliability of the regression results of the DID models, this article uses the PSM-DID method (42) proposed by Heckman et al. to philtre the control group through propensity score matching (PSM) and then uses the DID model for regression. Specifically, control variables are first used to predict the probability of each city becoming a pilot city for smart city construction. Then, we used the nearest neighbor-matching method to match the control group of the smart city construction pilot city so that there was no significant difference between the treatment group and the control group before the smart city construction policy was issued to reduce the endogeneity problems caused by self-selection bias during the smart city construction pilot. Figures 5, 6 are the kernel density maps of the propensity score before and after matching, respectively. The obtained results show that there is the significant closeness between the treatment group and the control group after the matching; thus, the control group samples obtained through propensity score matching are effective. Finally, the DID model is used to identify the impact of smart city construction on public occupational health quality. Because PSM can solve the deviation problem of observable covariables to the greatest extent, while the DID model can eliminate the influence of unobserved variables, such as constant and synchronous change with time, the combination of the two methods can better identify the impact of smart city construction on the quality of public occupational health.

Based on propensity score matching and balance tests, the impact of smart city construction on the quality of public occupational health was reestimated. As shown in Table 4, the coefficient of smart city construction is significantly positive at the 1% statistical level, indicating that the benchmark regression results are still valid after considering the selection bias.

**Table 4:** Results of the PSM+DID model.

	**ln_rate**	**ln_death**	**ln_rate**	**ln_death**
	**(1)**	**(2)**	**(3)**	**(4)**
Wise	−0.150***	−0.114**	−0.282***	−0.165***
	(0.056)	(0.050)	(0.057)	(0.053)
Control variables	No	No	Yes	Yes
Region FE	Yes	Yes	Yes	Yes
Year FE	Yes	Yes	Yes	Yes
Constant	0.359***	6.192***	7.030***	9.618***
	(0.056)	(0.050)	(1.119)	(1.052)
*R* ^2^	0.954	0.811	0.960	0.823
*N*	380	380	380	380

*** and **, respectively, indicate significance at the 1 and 5% level. Standard error in parentheses.

#### Falsification Tests

This article conducted a falsification test by changing the policy implementation time (43). In addition to the policy change of smart city construction, other policies or random factors issued by the Guangdong Province may also lead to changes in the quality of public occupational health, and such changes are not related to the construction of smart cities; thus, the conclusion is not valid. To eliminate the interference of such factors, the construction time of smart cities in each city is advanced uniformly by 1 year, 2 years, and 3 years and three hypothetical policy variables are constructed, including *Wise*__1_, *Wise*__2_, and *Wise*__3_. If the regression coefficients of the three hypothetical variables are significantly positive, it indicates that the improvement in the quality of public occupational health is likely to be caused by other policy changes or random factors rather than by the construction of smart cities. In contrast, if the regression coefficients of the three hypothetical variables are not significant, it indicates that the improvement of the quality of public occupational health quality comes from the policy implementation of smart city construction. Table 5 shows the regression results of smart city construction being artificially advanced by 3 years. The results show that the variable coefficient of hypothetical smart city construction is not significant, indicating that the improvement of the quality of public occupational health quality is, indeed, caused by smart city construction rather than other policies and factors.

**Table 5:** Results of falsification test.

	**ln_rate**	**ln_death**	**ln_rate**	**ln_death**
Wise_1	−0.121	−0.050	−0.091	−0.068
	(0.083)	(0.074)	(0.081)	(0.075)
Wise_2	0.046	−0.012	0.037	−0.009
	(0.107)	(0.095)	(0.101)	(0.093)
Wise_3	0.028	0.130*	0.021	0.124*
	(0.083)	(0.074)	(0.080)	(0.073)
Control variables	No	No	Yes	Yes
Region FE	Yes	Yes	Yes	Yes
Year FE	Yes	Yes	Yes	Yes
Constant	0.359***	6.192***	6.427***	9.732***
	(0.056)	(0.050)	(1.223)	(1.128)
*R* ^2^	0.954	0.811	0.958	0.821
*N*	380	380	380	380

*** and *, respectively, indicate significance at the 1 and 10% level. Standard error in parentheses.

#### Other Robustness Tests

We considered the impact of inherent city differences to obtain Table 6. Whether a city was identified as pilot smart city construction is closely related to the baseline factors, such as the location of the city, the existing economic and social development level, and the level of opening up. The existing differences between these cities affect the quality of public occupational health over time, resulting in biassed estimates. To control for the effects of these factors, the cross-term ($Z_{i}^{*}Trend_{t}$) of the baseline variable (*Z*_*i*_) and the time-linear trend (*Trend*_*t*_) are incorporated into the regression model to control the effect of the inherent differences between cities on the quality of public occupational health from a linear perspective. Based on Formula 1, this article constructed the following model (44).


(4) $$\begin{array}{r} Y_{it}=\beta_{0}+\beta_{1}Wise_{it}+\sum_{it}Control_{it}+Z_{i}\;*\;Trend_{t}+\mu_{i}+\delta_{i}\\ \;+\varepsilon_{it} \end{array}$$

where *Z*_*i*_ represents the geographical location of the city and its original political and economic characteristics, including whether it is a city in the Pearl River Delta region, whether it is a coastal city, and whether it is a special economic zone. *Trend*_*t*_ represents the time-linear trend. Therefore, $Z_{i}^{*}Trend_{t}\;$represents that the effect of the original inherent characteristic differences between cities on the quality of public occupational health is controlled from a linear perspective, and the estimation bias due to the nonrandom selection of treatment and control groups is mitigated to some extent again. Table 6 shows that the coefficient of smart city construction of all the models passed the significance level of at least 10%, indicating that the estimated results are still robust after considering the possible effects of inherent differences in cities.

**Table 6:** Results of controlling inherent differences of a city.

	**ln_rate**	**ln_death**	**ln_rate**	**ln_death**
	**(1)**	**(2)**	**(3)**	**(4)**
Wise	−0.124*	−0.120*	−0.173***	−0.122**
	(0.069)	(0.062)	(0.065)	(0.061)
Z*Trend_*t*_	Yes	Yes	Yes	Yes
Control variables	No	No	Yes	Yes
Region FE	Yes	Yes	Yes	Yes
Year FE	Yes	Yes	Yes	Yes
Constant	0.360***	6.192***	9.137***	11.988***
	(0.056)	(0.050)	(1.326)	(1.243)
*R* ^2^	0.954	0.811	0.962	0.830
*N*	380	380	380	380

***, **, and *, respectively, indicate significance at the 1, 5, and 10% level. Standard error in parentheses.

We control the impact of other guiding policies to obtain Table 7. If there are other policy shocks within the sample interval that are highly correlated with the explained variable, the accuracy of the estimates can also be affected. To eliminate the influence of other guidance policies, this article focuses on considering the influence of supervisory systems from the perspective of national-level guidance policies. The supervision system has been implemented since 2010, and, in the sample period of this article, six cities have been supervised by the central government due to serious production safety accidents. The supervision system may interfere with the impact of smart city construction on the quality of public occupational health. Based on Formula 1, this article constructs the following model:

(5) $$\begin{array}{r} Y_{it}=\beta_{0}+\beta_{1}Wise_{it}+\beta_{2}Super_{it}+\sum_{it}Control_{it}+\mu_{i}\\ \;+\delta_{i}+\varepsilon_{it} \end{array}$$

where *Super*_*it*_ represents whether city *i* was supervised in year *t*, and it takes one if city *i* was supervised in year *t* and the years after and zero otherwise. The data of the supervision system come from the official website of the Ministry of Emergency Management of China. As can be seen from the estimated results in Table 6, after controlling the influence of the supervision system, the effect of smart city construction on improving the quality of public occupational health is still significant.

**Table 7:** Results of controlling other policy effects.

	**ln_rate**	**ln_death**	**ln_rate**	**ln_death**
	**(1)**	**(2)**	**(3)**	**(4)**
Wise	−0.104*	−0.102*	−0.242***	−0.166***
	(0.057)	(0.052)	(0.058)	(0.055)
Super	Yes	Yes	Yes	Yes
Control variables	No	No	Yes	Yes
Region FE	Yes	Yes	Yes	Yes
Year FE	Yes	Yes	Yes	Yes
Constant	0.359***	6.192***	6.908***	10.455***
	(0.055)	(0.050)	(1.210)	(1.138)
*R* ^2^	0.955	0.811	0.961	0.825
*N*	380	380	380	380

*** and *, respectively, indicate significance at the 1 and 10% level. Standard error in parentheses.

We controlled the individual characteristics of officials to obtain Table 8. Party secretaries and mayors of both the cities are considered to have played an important role in improving the quality of public occupational health. This increases the number of observations relative to the previous observations. We collected the individual characteristics of party secretaries and mayors, including their age, tenure, education, and work experience characteristics (city, provincial, and central government departments). These data are mainly drawn from the “Party and Government Leading Cadre Database” and “Chinese Politicians Database,” and then we controlled the individual characteristics of officials for the impact of individual characteristics on the quality of public occupational health.

**Table 8:** Results of controlling the individual characteristics of officials.

	**ln_rate**	**ln_death**	**ln_rate**	**ln_death**
	**(1)**	**(2)**	**(3)**	**(4)**
Wise	−0.150***	−0.112***	−0.276***	−0.174***
	(0.040)	(0.036)	(0.040)	(0.038)
Control variables	No	No	Yes	Yes
Region FE	Yes	Yes	Yes	Yes
Year FE	Yes	Yes	Yes	Yes
Constant	0.095	6.227***	6.851***	10.711***
	(0.167)	(0.151)	(0.865)	(0.811)
*R* ^2^	0.954	0.812	0.961	0.826
*N*	760	760	760	760

***indicate significance at the 1% level. Standard error in parentheses.

### Heterogeneity Test

Hypothesis 2 assumes that the improvement effect of smart city construction on the quality of public occupational health may be affected by differences in administrative levels. This article constructs the interaction term of *Wise* and *SUB* variables to investigate the effect of administrative level differences on the impact of smart city construction on the quality of public occupational health. Among the 20 cities in the Guangdong Province, Guangzhou and Shenzhen are sub-provincial cities, while the other 18 cities are general prefecture-level cities. When city *i* is Guangzhou or Shenzhen, *SUB* is assigned to one; otherwise, it is assigned to zero. The result is obvious from Table 9. The coefficients of Wise^*^SUB are negative and pass the 1% significance level, which indicates that the effect of smart city construction on the quality of public occupational health is stronger in sub-provincial cities, that is, the smart city construction effect on the improvement of the quality of city public occupational health is affected by the administrative level difference. Hypothesis 2 is adopted.

**Table 9:** Results of different administrative levels.

	**ln_rate**	**ln_death**	**ln_rate**	**ln_death**
	**(1)**	**(2)**	**(3)**	**(4)**
Wise*SUB	−0.216**	−0.283***	−0.371***	−0.261***
	(0.100)	(0.090)	(0.098)	(0.093)
Control variables	No	No	Yes	Yes
Region FE	Yes	Yes	Yes	Yes
Year FE	Yes	Yes	Yes	Yes
Constant	0.359***	6.192***	7.340***	10.645***
	(0.055)	(0.049)	(1.186)	(1.118)
*R* ^2^	0.954	0.816	0.962	0.829
*N*	380	380	380	380

*** and **, respectively, indicate significance at the 1 and 5% level. Standard error in parentheses.

Hypothesis 3 assumes that the improvement effect of smart city construction on the quality of public occupational health may also be affected by regional differences. In this paper, the interaction term of *Wise* and *PRD* is used to investigate the effect of regional differences on the impact of smart city construction on the quality of public occupational health. According to the “Outline of the Reform and Development Plan for the Pearl River Delta region (2008–2020)” issued in 2009, this paper demarcates the regional scope. Nine cities, Guangzhou, Shenzhen, Zhuhai, Foshan, Jiangmen, Dongguan, Zhongshan, Huizhou, and Zhaoqing, are classified as PRD, and the remaining 12 cities are classified as non-PRD. When city *i* belongs to PRD, *RGO* is assigned a value of one; otherwise, it is assigned zero. As can be seen from the regression results of columns (1) and (3) in Table 10, the coefficients of Wise^*^RGO are negative and pass the significance level of 1%, indicating that the improvement effect of smart city construction on the quality of public occupational health is stronger in PRD. Namely, the improvement effect of smart city construction on the quality of public occupational health is affected by regional differences. Hypothesis 3 is also adopted.

**Table 10:** Results of different regions.

	**ln_rate**	**ln_death**	**ln_rate**	**ln_death**
	**(1)**	**(2)**	**(3)**	**(4)**
Wise*RGO	−0.421***	0.079	−0.408***	−0.024
	(0.150)	(0.135)	(0.147)	(0.138)
Control variables	No	No	Yes	Yes
Region FE	Yes	Yes	Yes	Yes
Year FE	Yes	Yes	Yes	Yes
Constant	0.359***	6.192***	8.071***	10.631***
	(0.056)	(0.050)	(1.233)	(1.165)
*R* ^2^	0.954	0.811	0.961	0.825
*N*	380	380	380	380

***indicate significance at the 1% level. Standard error in parentheses.

## Conclusions

In this article, based on the Guangdong Province of China from 2001 to 2018 panel data of 20 cities, fixed effects are constructed to realise the difference-in-difference model, which aims to accurately assess the impact of smart city construction on the quality of public health effects and further explore the effect of smart city construction on the quality of occupational health at different administrative levels and regions. The obtained results determined that smart city construction significantly improves the quality of public occupational health, which is still valid after the parallel trend test, falsification test, PSM-DID test, and a series of other robustness tests. The effect of smart city construction on improving public occupational health quality is affected by the difference in administrative levels. Compared with general prefecture-level cities, the effect of smart city construction on improving occupational health quality is stronger in sub-provincial cities. The effect of smart city construction on improving public occupational health quality is also affected by regional differences. Compared with the region of the non-Pearl River Delta, the effect of smart city construction on improving the quality of occupational health is stronger in the region of the Pearl River Delta.

Based on the results, this paper specifies the following policy implications. First of all, with the aim of strengthening the policy effect of smart city construction and improving the quality of public occupational health, all government departments should deepen the construction of smart cities and deeply understand the integration of smart city construction. Then, local governments should give full play to the role of public service providers, build technological innovation and research platforms, and provide good research innovation and research environment, which aims to ensure that the smart city pilot can provide the continuous technological impetus for improving public occupational health in a stable market environment. Finally, the central government should promote smart city construction according to local conditions, fully consider the administrative differences and regional differences of the city, and implement supporting policies to ensure the effective operation of smart city pilot mechanisms and the full release of policy dividends to effectively improve the quality of public occupational health.

## Data Availability Statement

The original contributions presented in the study are included in the article/supplementary material, further inquiries can be directed to the corresponding author/s.

## Author Contributions

HC: conceptualization and methodology. F-FW: writing and editing. D-WD: writing—original draft. J-CL: writing and reviewing. C-FZ: software and data preparation. BY: reviewing and proofreading. All authors contributed to the article and approved the submitted version.

## Funding

This research is partly supported by the Philosophy and Social Sciences Fund of Guangxi Province (20CJY001), Guangxi Higher Education Undergraduate Teaching Reform Project (2021JBG249), Entrusted Project by Information Centre of Guangxi Zhuang Autonomous Region (20210526), the National Social Science Fund of China (20CSH077), and Philosophy and Social Sciences Fund of Guangxi Province (20FMZ019).

## Conflict of Interest

C-FZ is employed by Nanning United Innovation Venture Capital Co., Ltd. The remaining authors declare that the research was conducted in the absence of any commercial or financial relationships that could be construed as a potential conflict of interest.

## Publisher's Note

All claims expressed in this article are solely those of the authors and do not necessarily represent those of their affiliated organizations, or those of the publisher, the editors and the reviewers. Any product that may be evaluated in this article, or claim that may be made by its manufacturer, is not guaranteed or endorsed by the publisher.
